# Attitudes towards persons with mental health conditions and psychosocial disabilities as rights holders in Ghana: a World Health Organization study

**DOI:** 10.1186/s12888-023-04620-3

**Published:** 2023-03-07

**Authors:** Briony Harden, Leveana Gyimah, Michelle Funk, Natalie Drew-Bold, Martin Orrell, Maria Francesca Moro, Celline Cole, Sally-Ann Ohene, Florence Baingana, Caroline Amissah, Joana Ansong, Priscilla Elikplim Tawiah, Kwaku Brobbey, Mauro Giovanni Carta, Akwasi Osei

**Affiliations:** 1 Institute of Mental Health, University of Nottingham, Innovation Park, Jubilee Campus, Triumph Road, Nottingham, NG7 2TU UK; 2 WHO Country Office for Ghana, Accra, GH Ghana; 3 Policy, Law and Human Rights, Department of Mental Health & Substance Use, World Health Organisation, Geneva, CH Switzerland; 4 Columbia Mailman School of Public Health, New York, NY USA; 5 Charité University Medicine Berlin, Berlin, DE Germany; 6 WHO Regional Office for Africa, Brazzaville, CG Congo; 7 Ghana Ministry of Health – Mental Health Authority, Accra, GH Ghana; 8 University of Cagliari, Cagliari, IT Italy

**Keywords:** Mental health, CRPD, Human rights, QualityRights, Attitudes, Rights-based care, World Health Organization, Coercion

## Abstract

**Background:**

There are currently major efforts underway in Ghana to address stigma and discrimination, and promote the human rights of those with mental health conditions, within mental health services and the community, working with the World Health Organization’s QualityRights initiative. The present study aims to investigate attitudes towards people with lived experience of mental health conditions and psychosocial disabilities as rights holders.

**Methods:**

Stakeholders within the Ghanaian mental health system and community, including health professionals, policy makers, and persons with lived experience, completed the QualityRights pre-training questionnaire. The items examined attitudes towards coercion, legal capacity, service environment, and community inclusion. Additional analyses explored how far participant factors may link to attitudes.

**Results:**

Overall, attitudes towards the rights of persons with lived experience were not well aligned with a human rights approach to mental health. Most people supported the use of coercive practices and often thought that health practitioners and family members were in the best position to make treatment decisions. Health/mental health professionals were less likely to endorse coercive measures compared to other groups.

**Conclusion:**

This was the first in-depth study assessing attitudes towards persons with lived experience as rights holders in Ghana, and frequently attitudes did not comply with human rights standards, demonstrating a need for training initiatives to combat stigma and discrimination and promote human rights.

## Introduction

In a population of 31 million [1], approximately 10% of Ghanaians live with a mental health condition or psychosocial, intellectual, and/or cognitive disability [2], and most are unable to access mental health care. Ghana has three public psychiatric hospitals with 1322 beds [3, 4], all in the south of the country [5], but there are 10 community inpatient psychiatric units [6]; however, these are all located towards the south of the country. Five of these are government-owned, whereas five are privately run (two in Accra and three in Kumasi) [6]. All district hospitals have mental health outpatient units, and ongoing work aims to integrate mental health into the community [6, 7]. Other private services are available but have little capacity and are too expensive for most people [5].

Within these mental health or related care services and the community, human rights violations occur, acting as major barriers to the delivery of good quality care, therefore hindering the recovery of persons with lived experience of mental health conditions or psychosocial disabilities [7]. From this point forwards, the term used will be “persons with lived experience”. Human rights are social, cultural, political, civil, and economic rights [8] that are fundamental to individuals and groups of people [9], including the right to an adequate standard of living for the health and wellbeing of the individual and the right to humane treatment [10]. In 2006, the United Nations adopted the Convention on the Rights of Persons with Disabilities (CRPD) [11]. The CRPD sets obligations on countries to promote and protect the rights of all persons with disabilities, including “persons with physical, mental, intellectual, or sensory impairments”. It is the first international human rights instrument which acknowledges that all persons with disabilities are rights holders and that a disability cannot justify denial or restrictions of human rights [9]. One of the key rights of the convention is that persons with disabilities have the right to equal recognition before the law, allowing them to exercise their legal capacity on an equal basis with others [11], meaning that persons with disabilities have the right to make their own decisions on all issues affecting them [12]. Article 14 promotes the right to liberty and security of a person, while CRPD Articles 15 and 16 state that persons with disabilities will not be subject to torture or cruel, inhuman, or degrading treatment or punishment (Article 15), and that they shall be free from exploitation, abuse, and violence (Article 16) [11]. Taken together, these rights safeguard against coercive practices including those experienced by many persons with lived experience, such as involuntary admission and treatment, as well as the use of seclusion and restraint. The CRPD has been ratified in many countries [13], including Ghana [14].

The Ghanaian mental health system has been appraised using the World Health Organization’s (WHO) Assessment Instrument for Mental Health Systems in both 2013 and 2020. Both appraisals found high rates of coercive practices in both formal and informal mental health services, illustrating severe rights violations [6, 7]. Coercive practices include the use of restraint, seclusion, substitute decision-making, and involuntary admission and/or treatment [12, 15–17]. In Ghanaian hospital settings, forced admission and treatment, restraint using mechanical, chemical, and manual means, and seclusion from other patients and staff were often reported [5–7, 18]. Within prayer camps and general health camps in the country, people were often subjected to chaining [19, 20] and forced admission and treatment [19, 21]. Furthermore, there was a severe lack of training for mental health workers on human rights [7].

Coercion is not unique to Ghana; these practices are in many countries and can be difficult for policy makers and services to change due to their general acceptance within mental health systems and society as a whole [22], often supported by restricted and outdated laws and policies. Additionally, laws that protect people’s rights are under-enforced in practice. Underlying attitudes also drive coercive practices [23] and mental health professionals, nurses, and the general public thinking coercive measures, particularly restraint, should be employed (especially with service users with severe mental health conditions) to maintain staff and patient safety [23–25]. These attitudes often lead to rights violations and can reinforce the idea that persons with lived experience are violent and dangerous individuals [26, 27], justifying coercive practices [28]. However, Vandamme et al. [29] found no relationship between implicit or explicit staff attitudes towards coercion and the use of coercion within psychiatric clinics. Nevertheless, there is increasing acknowledgement and evidence that coercive practices harm a person’s physical and mental health [13, 28, 30–32].

In many countries, including Ghana, persons with lived experience are often stigmatised and discriminated against due to a lack of understanding about mental health as well as societal norms and cultural beliefs; for example, that mental health conditions are caused by evil spirits, curses, or punishments from God for sinful behaviour [33–35]. People with lived experience can be seen as violent, dangerous, and unpredictable [27, 36, 37]. Because of this, they often experience inhumane treatments, exclusion from their community, and their families, and violence and neglect [37]. It can also lead to persons with lived experience not seeking help [38]. These coercive practices deny people’s basic human rights, something which over 40% of participants within Barke et al’s [36] study thought was acceptable. Overall, attitudes towards persons with lived experience tend to be negative [39], although this can depend on several factors, including demographics, levels of education, workplaces, and legislation [27, 38, 40–42].

There is an urgent need to implement human rights standards within mental health or related care, to ensure all persons with lived experience are free from coercion, are able to realise their legal capacity and right to decide over treatment, and are included within the community. In 2012, the Ghanaian government ratified the CRPD and passed the mental health act in an attempt to overhaul outdated legislation and to improve the quality of care and human rights within its mental health system. However, while the new Act aimed to protect human rights, improve access to care, and promote the recovery of persons with lived experience [43], it still permits for the use of involuntary treatment, substitute decision-making, and seclusion and restraint [44], allowing for human rights violations to continue. This is not in line with General Comment 1 of the UN Committee on the CRPD which provides guidance on the interpretation of Article 12 on equal recognition before the law [45]. Furthermore, a recent evaluation of psychiatric services within Ghana illustrated significant inadequacies in the implementation of the CRPD principles within practices [18], demonstrating a denial of rights. In 2019, the WHO’s QualityRights initiative was launched in the country [46] with the aim of transforming the mental health system towards a person-centred, rights-based approach.

### Quality rights initiative

The WHO developed the QualityRights initiative by building on the principles of the CRPD [11], with the intention of increasing access to better quality mental health and related care and promoting the rights of persons with lived experience [13]. In 2012, the newly published WHO QualityRights assessment toolkit offered practical guidance and tools to assist countries in evaluating and improving the quality of care and human rights standards within care systems [47]. Additionally, WHO QualityRights training modules on mental health, human rights, and recovery have been developed [48] in order to deliver face-to-face training, and more recently, online e-training. The training aims to enhance the understanding, implementation, and protection of human rights including within mental health services [48], addressing stigma and discrimination and improving attitudes towards persons with live experience [48]. The training was formulated through collaboration with many different expert groups who provided inputs at every stage of their development, including mental health professionals, people with lived experience, human rights experts, non-governmental organisations (NGOs), organisations of persons with disabilities (OPDs), policy makers and others [49].

### Quality rights initiative in Ghana

The activities of the initiative in Ghana include the assessment and transformation of mental health services, widescale training for primary healthcare prescribers in mental health, human rights, and recovery, and civil society engagement. Thus far, seven health facilities (including three mental health facilities and five general hospitals with mental health wings) have been assessed using the QualityRights assessment toolkit [18, 50] with service level transformation plans developed to address gaps identified in the assessment, which are soon to be implemented. The training has also been rolled out country-wide to key stakeholders including NGOs, persons with lived experience, persons working within both general and mental health or related sectors, and the general community [50]. As of the 13th September 2022, 22,091 people had completed the QualityRights e-training and received their certifications [50]. However, more work is still required to improve Ghana’s mental health and social care system.

Practices that violate human rights appear to be linked to the attitudes of those within mental health and related care, as well as to the stigma associated with mental health conditions and psychosocial disabilities, it is important to explore the attitudes towards persons with lived experience as rights holders. Whilst some research has examined stigma within the Ghanaian community [36, 37], there is a need for research to assess attitudes of stakeholders within health services and the wider community in relation to human rights perspectives.

### Aims and objectives

The current study aims to explore attitudes towards persons with lived experience as rights holders, including attitudes towards coercion, legal capacity, service environment, treatment choice and hope, and community inclusion.

## Methods

This research was undertaken as part of a larger project by the WHO, Mental Health Authority Ghana, and several other partners, which aimed to explore the impact of the QualityRights capacity building activities on attitudes and practices towards persons with lived experience as rights holders and used data collected from the pre-training assessments between February 2019 and June 2021.

### Design

Several key stakeholder groups within the Ghanaian mental health sector oversaw the QualityRights initiative and collaborated to promote the e-training across the country and encourage as many people as possible to complete the training. These groups include the WHO Ghana, Mental Health Authority Ghana, Ghana Health Service, Mental Health Society of Ghana, MindFreedom Ghana, BasicNeeds Ghana, Inclusion Ghana, Ta-Excel Foundation, Passion for Total Care, Special Olympics, and the Christian Health Association of Ghana.

The pre-training questionnaire included items assessing the attitudes towards coercion, legal capacity, service environment, treatment choice and hope, and community inclusion. Demographic factors included age, gender (male/female/prefer not to say/other), previous background/experience (Table 1), and current affiliation (Table 2). For the purposes of this research, academic/education experience includes students, teachers, and educators.

**Table 1:** Background/experience groupings

Groups	N	%
Healthcare professionals	2744	76.2
Health practitioner		
Mental health or related practitioner		
Social/Legal work	237	6.6
Human rights advocate		
Lawyer		
Policy maker/analyst		
Social worker		
Academics/Education	573	15.9
Academic		
Student		
Teacher		
Personal Experience*	361	10.0
Family member or care partner	155	4.3
Person with lived experience of mental health conditions	173	4.8
Person with other disabilities	62	1.7
Other Experience	381	10.6
Administrator/Manager		
Other		

*groups analysed separately due to significantly different types of personal experience

### Participants

The participants were key stakeholders within Ghana’s mental health system and the community more broadly. These stakeholders include both general health and mental health and related care professionals, persons with lived experience and their caregivers, NGOs, and members of the community more broadly, such as secondary school students. Participants with lived experience were contacted mainly through NGOs and Organisations of Persons with Disabilities. Interested persons could sign up for the e-training programme and to gain access to the modules, were requested to first complete the pre-training questionnaire.

### Ethical approval

The WHO study gained ethical approval from the Ghana Health Service Ethics Review Committee (GSH-ERC 001/09/19), and approval by the Independent Ethics Committee at the University Hospital of Cagliari.

Participants were presented with a consent form on the QualityRights e-learning platform which detailed the aims of the training, informed the participants that their participation was voluntary, and they could interrupt their training at any point. The consent form further indicated they could withdraw themselves and their data at any time. The data were kept confidential in accordance with the provisions that protect privacy in Ghana (Data Protection Act 2012), and Articles Six and Nine of the EU Regulation.

### Measures

The questionnaire [51] was developed specifically for the WHO QualityRights training by the WHO’s QualityRights team, and consisted of 17-items (Table 3) relating to attitudes towards persons with lived experience as rights holders. The items can be grouped into four themes: (1) Coercion; (2) Legal Capacity; (3) Service environment, treatment choice, and hope; and (4) Community Inclusion (Table 3). The statements are answered using a 5-point Likert-scale (1 = strongly disagree to 5 = strongly agree). All bar three statements were negatively worded, with items G, M, and Q (Table 3) reverse-scored. The highest score possible overall was 85, the lowest possible was 17. Higher scores indicate attitudes towards persons with lived experience as rights holders that do not comply with human rights standards. The questionnaire had good internal consistency for the sample of the current study (α = 0.75).

### Procedure

Once participants had registered their interest for the e-training, they were presented with the consent form. After signing this, they completed demographic questions, before moving onto the attitudinal items.

### Analysis

The analyses were conducted on IBM SPSS Statistics v27 [52]. Thorough data cleaning took place before analyses were conducted. Any missing data were automatically excluded when analyses were completed. Demographics included current affiliations, and background/experience. Participants chose their current affiliations from a drop-down menu of 13 options (Table 2). Background/experiences and additional background/experiences were chosen from two standardised drop-down menus which included the same 11 options (Table 1). Additionally, many participants who chose ‘other’ were students or teachers, so these categories were included. These backgrounds were grouped into five broad categories (Table 1). As the participants could choose two different backgrounds, it meant they could be included within up to two of the groupings. Doing this allowed for direct comparisons between participants with certain backgrounds and those without. These comparisons were made using independent-samples t-tests.

The attitudes investigated were the overall participant attitudes and attitudes by affiliations (Table 3) using descriptive analyses, whilst each individual statement was analysed using the t-tests to explore potential differences between background/experience groups and their attitudes.

## Results

Overall, 4090 participants responded to the survey. As 491 did not consent to their data being used in research, the final full sample was 3599 (1907 male). The mean age of the participants was 30.51 (6.23).

### Overall attitudes

Table 3 shows the spread of answers for each item on the questionnaire from the 3599 participants. The statements are ordered according to which items demonstrate the attitudes that do not comply with a human rights-based approach to mental health first. On the whole, attitudes towards persons with lived experience as rights holders were not aligned with a human rights framework.

**Table 2:** Attitude scores by affiliation (higher score = more negative attitudes)

Affiliation	N (total = 3555)	Mean score (standard deviation)
Unknown	47	53.45 (6.53)
Academia	364	49.05 (8.31)
Other Governmental Ministry/Department/Commission	85	48.95 (9.62)
Disabled People’s Organisation	24	48.71 (8.82)
Other	130	48.35 (9.10)
Donor/Funder	3	48.00 (10.54)
Non-governmental organisations	205	48.00 (8.51)
Professional organisation/association	58	47.62 (7.53)
Service provider (general health)	200	46.79 (8.82)
Ministry of Health	1855	46.74 (8.84)
UN organisation and agencies	4	45.50 (5.75)
Service provider (mental health or related areas	575	44.17 (8.35)
Multilateral organisation or development agency	5	42.80 (2.05)

**Table 3:** Attitudes to statements on mental health and human rights

Statement	Strongly agree/agree	Neutral	Strongly disagree/disagree
O. The use of seclusion and restraint is needed if people using mental health services become threatening^a^	70.91	9.64	19.45
L. When people experience a crisis, health practitioners or families should make decisions based on their ideas about what is best for them^b^	60.93	10.59	28.48
*Q. Involuntary admission does more harm than good^a^	30.92	20.64	48.44
N. Controlling people using mental health services is necessary to maintain order^a^	46.85	15.28	37.87
I. The opinions of health practitioners about care and treatment should carry more weight than those of a person with an intellectual disability^b^	46.60	16.61	36.79
C. People with dementia should always live in group homes where staff can take care of them^d^	45.60	11.75	42.65
P. People at risk of harming themselves or others should be isolated in a locked room^a^	44.01	10.31	45.68
E. Taking medication is the most important factor to help people with mental health conditions get better^c^	42.96	11.64	45.40
A. Nothing can be improved within mental health services without additional resources^c^	40.12	7.42	52.46
F. You can only inspire hope once a person is no longer experiencing symptoms^c^	29.15	9.70	61.15
*M. People with intellectual disabilities have the right to make their own decisions, even if I don’t agree with them^b^	61.82	13.70	24.48
B. The service environment has little to do with people’s mental health and wellbeing^c^	24.23	6.75	69.02
*G. People using mental health services should be empowered to make their own decisions about their own treatment^c^	68.72	9.97	21.31
D. People with psychosocial disabilities/mental health conditions should not be hired in work requiring direct contact with the public^d^	17.83	9.03	73.14
K. People with mental health conditions should not be given important responsibilities^b^	15.81	10.78	73.41
H. Following advice of other people who have experienced mental health issues is too risky^b^	15.42	16.20	68.38
J. It is acceptable to pressure people using mental health services to take treatment that they don’t want^a^	14.86	9.39	75.75

*reverse scored, ^a^coercion, ^b^legal capacity, ^c^service environment, treatment choice, and hope, ^d^community inclusion

### Coercion

Overall, attitudes towards coercion were not aligned with a human rights framework. Most people agreed with the use of coercive techniques within treatment, including the use of restraint and seclusion (70.91%), while many believed that involuntary admission was more beneficial than harmful (48.43%) and that controlling people to maintain order was acceptable (46.85%). However, only 14.86% agreed that it was acceptable to pressure people into unwanted treatment.

#### Healthcare professionals

Compared to other groups, people with healthcare professional experience: were less likely to think it was acceptable to pressure people using mental health services into treatment, mean difference (MD)=-0.62, BCa 95% CI [-0.696,-0.542], t(1056.79)=-14.999, p = .000, d = 0.67; were less likely to think that controlling people was necessary to maintain order within mental health services, MD=-0.26, BCa 95% CI [-0.354,-0.170] t(1479.33)=-5.64, p = .000, d = 0.22; and less likely to believe that involuntary admission did more harm than good, MD = 0.37, BCa 95% CI [0.287,0.455], t(1497.09) = 8.54, p = .000, d = 0.33.

#### Social/legal work

Compared to other groups, participants with experience in social/legal work were: more likely to think it was acceptable to pressure people using mental health services to undergo treatment they do not want to have, MD = 0.42, BCa 95% CI [0.282,0.571], t(249.27) = 5.66, p = .000, d = 0.46; they were also more likely to think that involuntary admission did more harm than good, MD=-0.23, BCa 95% CI [-0.395,-0.076], t(3597)=-2.98, p = .003, d = 0.20.

#### Academics/education

People with academic backgrounds, including teaching, were more likely to think that involuntary admission does more harm than good compared to other groups, MD=-0.35, BCa 95% CI [-0.446,-0.264], t(842.02)=-7.10, p = .000, d = 0.31.

#### Personal experience

##### Family member or care partner

Compared to other groups, family members or care partners of persons with lived experience were: more likely to think it was acceptable to pressure people using mental health services into unwanted treatment, MD = 0.66, BCa 95% CI [0.460,0.847], t(158.87) = 6.79, p = .000 d = 0.68; and also more likely to think that it was necessary to control people to maintain order, MD = 0.263, BCa 95% CI [0.083,0.434], t(3597) = 2.65, p = .008, d = 0.22.

##### Person with lived experience

Compared to other groups, participants with lived experience were more likely to think that the use of seclusion and restraint was needed if people using mental health services become threatening, MD = 0.211, BCa 95% CI [0.176,0.248], t(3293.00) = 11.42, p = .000, d = 0.28.

##### Person with other disabilities

Compared to other groups, people with other disabilities were: more likely to think it was acceptable to pressure people into unwanted treatment, MD = 0.45, BCa 95% CI [0.183,0.742], t(61.84) = 3.13, p = .003, d = 0.49; more likely to think controlling people using mental health services was necessary to maintain order, MD = 0.65, BCa 95% CI [0.344,0.922], t(62.11) = 5.08, p = .000, d = 0.59; more likely to think seclusion and restraint was needed if people become threatening, MD = 0.444, BCa 95% CI [0.284,0.627], t(52.97) = 5.42, p = .000, d = 0.52; and more likely to think involuntary admission did more harm than good, MD=-0.303, BCa 95% CI [-0.589,-0.011], t(3597)=-2.05, p = .041, d = 0.26.

#### Other experience

Those with administrative/managerial backgrounds and other experiences were more likely to agree that it was acceptable to pressure people using mental health services to take treatment they did want than those without these backgrounds, MD = 0.55, BCa 95% CI [0.432,0.662], t(414.02) = 9.10, p = .000, d = 0.59.

### Legal capacity

Most participants thought that persons with lived experience should not make their own decisions when in crisis (60.93%) and that the opinions of professionals should mean more than those with intellectual disabilities (46.60%). However, 61.82% agreed that persons with lived experience have the right to make decisions, suggesting a difference between attitudes towards general decision-making and attitudes towards decision-making when in crisis. Furthermore, many felt that people with intellectual disabilities should be empowered to make their own decisions (68.72%).

#### Healthcare professional

Compared with other groups, people with healthcare professional experience were less likely to think that it was risky to follow the advice of persons with lived experience of mental health issues, MD=-0.20, BCa 95% CI [-0.268,-0.134], t(1344.72)=-5.67, p = .000, d = 0.23; and were less likely to think that people with lived experience should not be given important responsibilities, MD=-0.63, BCa 95% CI [-0.708,-0.565], t(1029.82)=-16.41, p = .000, d = 0.67.

#### Academics/education

Compared to other groups, those with academic and educational backgrounds were more likely to think that people with mental health conditions should not be given important responsibilities, MD = 0.55, BCa 95% CI [0.468,0.645], t(645.29) = 11.96, p = .000, d = 0.63.

#### Personal experience

##### Family member or care partner

Compared to other groups, family members or care partners of persons with lived experience were: less likely to think following the advice of individuals with mental health conditions was too risky, MD=-0.25, BCa 95% CI [-0.277,-0.219], t(3328.00)=-16.09, p = .000, d = 0.40; more likely to think people with mental health conditions should not be given important responsibilities, MD = 0.255, BCa 95% CI [0.074,0.442], t(161.82) = 2.64, p = .005, d = 0.22; and less likely to think that people with intellectual disabilities have the right to make their own decisions, MD = 0.267, BCa 95% CI [0.095,0.451], t(163.88) = 2.71, p = .008, d = 0.24.

##### Person with lived experience

Compared to other groups, people with lived experience were: less likely to think people with mental health conditions should not be given important responsibilities, MD=-0.363, BCa 95% CI [-0.471,-0.265], t(191.66)=-6.54, p = .000, d = 0.40; and more likely to think that in a crisis, health practitioners and family members should make decisions based on their ideas of what is best for people with mental health conditions, MD = 0.35, BCa 95% CI [0.172,0.514], t(183.12) = 4.28, p = .000, d = 0.33.

##### Person with other disabilities

Compared to other groups, people with other disabilities were more likely to think that following the advice of people with experience of mental health issues was too risky, MD = 0.36, BCa 95% CI [0.077,0.669], t(62.21) = 2.34, p = .023, d = 0.34.

### Service environment, treatment choice, and hope

Medication was believed by many participants as the most important factor to help persons with mental lived experience recover (42.96%). Over 47% either agreed or had no opinion that nothing can be improved within mental health or related care services without additional resources. Whereas 38.84% believed, or did not know whether, hope could only be instilled once no symptoms were experienced. Over 30% either disagreed with, or held no attitude about, people using mental health services being empowered to make decisions about their own treatment. Furthermore, just under 25% agreed that the service environment has nothing or little to do with the mental health and wellbeing of service users.

#### Healthcare professional

Compared to other groups, those with healthcare professional were more likely to disagree that nothing can be improved within mental health services without additional resources, MD=-0.27, BCa 95% CI [-0.371,-0.181], t(3597)=-5.16, p = .000, d = 0.20, and were more likely to think that people using mental health services should be empowered to make their own decisions about their own treatment, MD=-0.35, BCa 95% CI [-0.453,-0.260], t(1346.87)=-7.47, p = .000, d = 0.29.

### Community inclusion

Attitudes towards the community inclusion of persons with lived experience were, on the whole, negative. Over 45% of participants believed that people with dementia should not live independently, and instead should live in group homes where staff can care for them. Additionally, 17.83% of participants felt that persons with lived experience should not be employed in work requiring direct contact with the public.

#### Healthcare professional

Compared to other groups, people with healthcare professional experience were less likely to think that people with dementia should always live in group homes where staff could look after them, MD = 0.26, BCa 95% CI [-0.353,-0.176], t(1484.13)=-5.38, p = .000, d = 0.21, and were less likely to think that people with mental health conditions should not be hired in work requiring direct contact with the public, MD=-0.99, BCa 95% CI [-1.089,-0.904], t(1019.05)=-22.22, p = .000, d = 0.99.

#### Academics/education

Those with an academic/educational background were more likely to think that people with mental health conditions should not be employed in work which requires direct contact with the public compared to other groups, MD = 0.84, BCa 95% CI [0.739,0.984], t(641.98) = 15.63, p = .000, d = 0.84.

#### Personal experience

##### Family member or care partner

Family members or care partners of persons with lived experience were more likely to think that people with mental health conditions should not be employed in work requiring direct contact with the public compared to other groups, MD = 0.32, BCa 95% CI [0.137,0.506], t(3597) = 3.34, p = .001, d = 0.27.

##### Person with lived experience

Compared to other groups, people with lived experience were: more likely to think people with dementia should live in group homes where they can be taken care of by staff, MD = 0.29, BCa 95% CI [0.108,0.474], t(3597) = 2.94, p = .003, d = 0.23; and less likely to think that people with mental health conditions should not be employed in work that requires direct contact with the public, MD=-0.45, BCa 95% CI [-0.552,-0.330], t(201.42)=-8.22, p = .000, d = 0.48.

##### Person with other disabilities

People with other disabilities were more likely to think that people with dementia should always live in group homes where staff can look after them compared to other groups, MD = 0.38, BCa 95% CI [0.056,0.675], t(3597) = 2.30, p = .022, d = 0.29.

## Discussion

This was the first in-depth study of attitudes in Ghana towards persons with lived experience as rights holders. On the whole, many participants agreed with the use of coercive techniques within mental health and related care, and that health practitioner’s opinions should carry more weight than those of persons with lived experience, especially when people with such conditions are in crisis. This was in spite of many participants believing people should be empowered to make their own treatment decision. Paradoxically, respondents on the training questionnaire may be demarcating on questions regarding decisions; they are agreeing, overall, that persons with lived experience should have the right to make their own decisions, but not when they are experiencing a crisis when their capacity is compromised. This may be based on an assumption that people in crisis are incapable of knowing what they want and decisions, therefore, have to be made for them regardless of their will and preference.

Regarding social factors, across all attitudinal themes, participants with healthcare professional experience held attitudes which were more compliant with human rights standards than those without this experience, mostly disagreeing with coercive measures and agreeing that people with lived experience should be able to choose their treatment and be involved in the community. Overall, people with personal experience of mental health conditions and services were often supportive of the use of seclusion, restraint, control, and involuntary treatments compared to other groups. This could be due to self-stigma, and the internalisation of the stigmatising attitudes and beliefs of people close to them as well as reflecting wider prevailing community attitudes [33]. Participants with lived experience were also more likely to agree with not including people with dementia in the community, stating they should be cared for in group homes. In contrast, persons with lived experience were more likely to think people with mental health conditions should be given important responsibilities and should be employed in work involving direct contact with the public. These suggest conflicting attitudes towards the rights of people with different mental health conditions. It may be that people assume that the capacity and capabilities of those living with dementia are significantly deteriorated compared to people with other mental health conditions. It may also be considered that Ghana has little to no facilities for the social support and health care of people living with dementia within the community [5]; thus group homes where staff can take care of them might be considered the most appropriate option.

### Previous research and practices

Past research conducted within Sub-Saharan Africa has reported high levels of stigma in the area of mental health [32, 42], which this study corroborates. Many participants within Barke et al.’s [36] study in Southern Ghana agreed that persons with lived experience should be isolated away from the general community and should not be given responsibility, which the present study also identified. Additionally, over 40% participants did not see a problem with denying the rights of persons with lived experience [36], mirroring the results of this study. Furthermore, research from other countries has reported that health professionals recommend coercion, especially restraint and seclusion, when working with persons with lived experience [23, 24]. These results support that the claim by Morandi; that the coercive and inhumane practices are the result of the stigmatising attitude that people with mental health conditions are dangerous, unpredictable, and violent [23].

Moro et al. (2022) completed a recent evaluation of several mental health facilities within Ghana and identified that many of the CRPD principles had not been implemented within their practices [18]. The rights to freedom from torture or cruel, inhuman or degrading treatment or punishment and from violence, exploitation, and abuse were violated within all psychiatric facilities evaluated [18]. The findings of previous research and the present investigation stress the importance of movements, actions, and initiatives, such as the WHO QualityRights initiative, to improve attitudes and challenge stigma profoundly and sustainably towards mental health and persons lived experience as rights holders.

### Strengths and limitations

This study was important due to the paucity of research on stigma, discrimination, and human rights violations. A major strength of this study was the use of an online questionnaire. This cost-effective method of data-collection allowed the researchers to obtain data from several thousands of participants, and from a diverse group of people thanks to its widespread dissemination among several community groups and on social media platforms. Furthermore, as the questionnaire was provided online, participants might have provided more truthful answers due to being away from the oversight of the face-to-face trainers, and therefore might not feel pressured into providing socially desirable answers. In addition, the current study highlighted which stakeholders hold more negative attitudes, such as those with personal experiences, academic/educational groups, and not healthcare professionals.

However, this study is not without limitations. Despite the dissemination of the e-training to the widely among the Ghanaian population, the majority of the sample in this research were general health or mental health and related care professionals, thus the results are not representative of the Ghanaian population. As the programme continues to be disseminated through a variety of methods, more people will be reached to obtain a more generalisable sample. Moreover, although significant differences were found between different demographic groups regarding attitudes, in some cases the effect sizes were not large. Hence, some of these results may be less meaningful and could be due to a type 1 error.

Furthermore, in some areas of affiliation, it was difficult to get a clear picture of the differences in attitudes between groups. For example, many people working for the Ministry of Health could be service providers of either general health or mental health services. This means there may be variations within this group that we are not able to explore with the available data.

### Future research and practical implications

Further work will evaluate the impact of the QualityRights e-training on attitude change, as well as the impact of the more intensive face-to-face training which has also been implemented. In addition, work is underway to develop improvement/transformation plans for the mental health facilities, as well as to evaluate the impact of such plans on service delivery. Practical implications of the current study include those for the QualityRights initiative and implications for policy makers within mental health care. The research demonstrates the need for the QualityRights initiative in Ghana to continue and reach as many people within the population as possible. The results of this study can also inform the Ghanaian partners about the current attitudes held, helping to direct future efforts to address stigma, combat discrimination, and promote rights. Further research could also be to determine whether the issues discussed in the study are reflective of long-held cultural and societal beliefs, or are the result of imported models of mental health from the West. It is challenging to disentangle the complexity of these issues. Finally, this research allows for comparative studies between countries to take place.

## Conclusion

This study was the first study to examine pre-training attitudes towards persons with lived experience as rights holders within Ghana. On the whole, participant’s agreed with the use of coercion and with imposing restrictions on the exercise of legal capacity for people with such conditions. Attitudes were often non-compliant with human rights frameworks. Participants with academic/educational experience, and those with personal experience of mental health conditions often had more negative attitudes.

The current research demonstrates the need to improve attitudes towards persons with lived experience in Ghana as rights holders, and it signifies the importance of the QualityRights initiative to reduce stigma and discrimination, and improve mental health services and human rights, within the country.

## Data Availability

The dataset used and analysed throughout the study is available from MF (funkm@who.int) or MGC (maurogcarta@gmail.com) on reasonable request.
